# New records of Helophoridae, Hydrochidae, and Hydrophilidae (Coleoptera) from New Brunswick, Canada

**DOI:** 10.3897/zookeys.573.7020

**Published:** 2016-03-24

**Authors:** Reginald P. Webster, Jon D. Sweeney

**Affiliations:** 1 24 Mill Stream Drive, Charters Settlement, NB, Canada E3C 1X1; 2 Natural Resources Canada, Canadian Forest Service - Atlantic Forestry Centre, 1350 Regent St., P.O. Box 4000, Fredericton, NB, Canada E3B 5P7

**Keywords:** Helophoridae, Hydrochidae, Hydrophilidae, Hydrophilinae, Chaetarthriinae, Enochrinae, Sphaeridiinae, new records, Canada, New Brunswick

## Abstract

The following three species of Helophoridae are newly recorded for New Brunswick, Canada: Helophorus (Kyphohelophorus) turberculatus Gyllenhal, Helophorus (Rhopaleloporus) oblongus LeConte, Helophorus (Rhopaleloporus) marginicollis Smetana. *Hydrochus subcupreus* Randall, family Hydrochidae, and the following 15 species of Hydrophilidae are newly reported for the province: *Berosus fraternus* LeConte, *Berosus peregrinus* (Herbst), *Berosus sayi* Hansen, *Paracymus despectus* (LeConte), *Chaetarthria atra* (LeConte), *Cymbiodyta acuminata* Fall, *Cymbiodyta blanchardi* Horn, *Cymbiodyta minima* Notman, Enochrus (Lumetus) hamiltoni Horn, Enochrus (Methydrus) consors (LeConte), Enochrus (Methydrus) consortus Green, Enochrus (Methydrus) pygmaeus nebulosus (Say), Cercyon (Cercyon) cinctus Smetana, Cercyon (Cercyon) herceus frigidus Smetana, Cercyon (Dicyrtocercyon) ustulatus (Preyssler).

## Introduction

This paper treats new records of Helophoridae, Hydrochidae, and Hydrophilidae from New Brunswick, Canada. A few brief comments are required regarding the family status of Helophoridae, Hydrochidae, and Georissidae as there has been some disagreement in the literature. Smetana (1988) in the review of the Hydrophilidae of Canada and Alaska treated the Helophorinae and Hydrochinae as subfamilies of the Hydrophilidae. Van Tassel (2000) provided a general overview on the taxonomy and classification of North American members of the Hydrophilidae and also treated these taxa as subfamilies of the Hydrophilidae. This arrangement was subsequently used by Bousquet et al. (2013) in the Checklist of the Beetles of Canada. However, Hansen (1991), employing adult characters, and Archangelsky (1998), using adult and larval characters, provided data that support treating these subfamilies as families (Helophoridae, Hydrochidae, Georissidae). See Van Tassel (2000) for more details and references regarding this issue. Recently, Short and Fikáček (2013) provided a revised classification of the Hydrophilidae based on morphological data and molecular analysis of DNA sequence data from mitochondrial and nuclear genes. They also considered the Helophoridae, Hydrochidae, and Georissidae as separate families and made a number of changes to the classification of the Hydrophilidae. We follow their classification in this publication.

The Helophoridae and Hydrochidae are aquatic and usually occur in fresh water (Smetana 1988, Van Tassel 2000). The Hydrophilidae (water scavenger beetles) occurring in Canada can generally be divided into two biologically different groups, one aquatic and the other terrestrial, but see Short and Fikáček (2013) and Bloom et al. (2014) for more detailed discussions on the relationship between diversity patterns, habitat associations, and taxonomic lineages in this family from a global perspective. The aquatic species, which are the most species rich in Canada, belong to the subfamilies Hydrophilinae, Chaetarthriinae, and Enochrinae (Smetana 1988, Van Tassel 2000, Short and Fikáček 2013). Members of the Sphaeridiinae are mostly terrestrial, with a few semi-aquatic species. Aquatic species usually live in stagnant pools, littoral areas of lakes and ponds, in shallow water of streams, and in springs, and a few occur in brackish to strongly saline water (Smetana 1988, Van Tassel 2000). Immature stages are predaceous, and adults are mostly omnivorous, feeding on decaying vegetation and plants, but some are predatory on snails and other small invertebrates (Van Tassel 2000). The terrestrial species are scavengers in fresh mammal dung, soil rich in humus, rotting mushrooms, seaweed, or among moist decaying leaves (Smetana 1988, Van Tassel 2000). See Van Tassel (2000) for further details on the biology of North American members of this family.

Thirty-eight species of Hydrophilidae, including the Helophoridae and Hydrochidae, were reported for New Brunswick by Smetana (1988) and Roughley (1991). Since those publications, little has been published on the Hydrophilidae of New Brunswick. In the most recent checklist of the beetles of Canada, eight species of Helophoridae (as Helophorinae), one species of Hydrochidae (as Hydrochinae), and 37species of Hydrophilidae were reported for the province (Bousquet et al. 2013). During a general survey of the Coleoptera of New Brunswick, an additional three species of Helophoridae, one species of Hydrochidae, and 15 species of Hydrophilidae have been recorded for the province. The purpose of this paper is to report on these new records.

## Methods and conventions

**Collection methods.** The following records are based, in part, on specimens collected as part of a general survey to document the Coleoptera fauna of New Brunswick. Helophoridae, Hydrochidae, and Hydrophilidae were sampled from various aquatic and semi-aquatic habitats. Aquatic habitats were sampled with aquatic nets. Very small aquatic and semi-aquatic habitats, such as vernal pools, spring-fed seepages, and moss and debris on stream margins, were sampled by removing moss and debris and placing it on a cloth sheet or aquatic net to drain water away. The specimens were sifted and collected as they became active. Some specimens were collected from Lindgren funnel trap samples during a study to develop methods for improved survey and detection of potentially invasive species of bark and wood-boring beetles. These traps are visually similar to tree trunks and are often effective for sampling species of Coleoptera that live in microhabitats associated with standing trees (Lindgren 1983), but species associated with other habitats are often collected as well. At many sites, equal numbers of traps were deployed in the canopy and 1 m high under trees. Traps were baited with various combinations of lures for detecting Cerambycidae. See Webster et al. (2012) for details of the lures used and Hughes et al. (2014) for methods used to deploy Lindgren traps and sample collection. A description of the habitat was recorded for all specimens collected during this survey. Locality and habitat data are presented as on labels for each record. Two labels were used on many specimens, one that included the locality, collection date, and collector, and one with macro- and microhabitat data and collection method. Information from the two labels is separated by a // in the data presented from each specimen.

**Specimen preparation.** Males of Hydrochidae and some species of Helophoridae and Hydrophilidae were dissected to confirm their identity. The genital structures were dehydrated in absolute alcohol and mounted in Canada balsam on celluloid microslides and then pinned with the specimens from which they originated. Keys in Smetana (1985 and (1988) were used to determine specimens.

**Distribution.** Every species is cited with current distribution in Canada and Alaska, using abbreviations for the state, provinces, and territories. New records for New Brunswick are indicated in bold under Distribution in Canada and Alaska. The following abbreviations are used in the text:

AB
 Alberta 


AK
 Alaska 


BC
 British Columbia 


MB
 Manitoba 


NB
 New Brunswick 


NF & LB
 Newfoundland and Labrador* 


NS
 Nova Scotia 


NT
 Northwest Territories 


NU
 Nunavut 


ON
 Ontario 


PE
 Prince Edward Island 


QC
 Quebec 


SK
 Saskatchewan 


YT
 Yukon Territory 


*Newfoundland and Labrador are each treated separately under the current Distribution in Canada and Alaska.

Acronyms of collections examined or where specimens reside referred to in this study are as follows:

AFC
 Atlantic Forestry Centre, Fredericton, New Brunswick, Canada 


NBM
 New Brunswick Museum, Saint John, New Brunswick, Canada 


RWC
 Reginald P. Webster Collection, Charters Settlement, New Brunswick, Canada 


## Results

### Species accounts

All records below are species newly recorded for New Brunswick, Canada. Species with a † are adventive to Canada, species with a * are Holarctic. The determination that a species was a new record was based on information in the print version of Bousquet et al. (2013). The classification used below follows Short and Fikáček (2013).

### Family Helophoridae Leach, 1815

#### Helophorus (Kyphohelophorus) tuberculatus

Taxon classificationAnimaliaColeopteraHydrophilidae

Gyllenhal, 1808*

##### Material examined.

**New Brunswick, Restigouche Co.**, near Little Tobique River, 47.4503°N, 67.0583°W, 13.VI.2006, R.P. Webster // Eastern white cedar swamp, in saturated moss in small pool (1, RWC).

##### Distribution in Canada and Alaska.

AK, YT, BC, AB, SK, MB, ON, QC, **NB**, NS (Bousquet et al. 2013).

#### Helophorus (Rhopalelophorus) marginicollis

Taxon classificationAnimaliaColeopteraHydrophilidae

Smetana, 1985

##### Material examined.

**New Brunswick, Madawaska Co.**, 4.0 km W of Saint-Hilaire, 47.2926°N, 68.4622°W, 27.VII.2006, R.P. Webster // Margin of Saint John River in rock pool (1, RWC).

##### Distribution in Canada and Alaska.

ON, **NB** (Bousquet et al. 2013).

#### Helophorus (Rhopalelophorus) oblongus

Taxon classificationAnimaliaColeopteraHydrophilidae

LeConte, 1850

##### Material examined.

**New Brunswick, Restigouche Co.**, Morin Bog, N of Kedgwick, 47.6813°N, 67.3142°W, 22.V.2003, R.P. Webster // Black spruce forest, flooded semi-permanent sedge marsh [pond] (1, RWC).

##### Distribution in Canada and Alaska.

AK, YT, NT, NU, BC, AB, SK, MB, ON, QC, **NB** (Bousquet et al. 2013).

### Family Hydrochidae C.G. Thomson, 1859

#### Hydrochus subcupreus

Taxon classificationAnimaliaColeopteraHydrophilidae

Randall, 1838

##### Material examined.

**New Brunswick, York Co.**, Charters Settlement, 45.8395°N, 66.7391°W, 26.VI.2003, 17.VII.2004, 1.VIII.2004, 11.VI.2005, 10.VII.2005, 20.VII.2006, 3.IX.2010, R.P. Webster // Mixed forest, m.v. light (10 [6 males dissected], RWC)

##### Distribution in Canada and Alaska.

ON, QC, **NB** (Bousquet et al. 2013).

### Hydrophilidae, Latreille, 1802

#### Subfamily Hydrophilinae Latreille, 1802

##### Tribe Berosini Mulsant, 1844

###### Berosus fraternus

Taxon classificationAnimaliaColeopteraHydrophilidae

LeConte, 1855

####### Material examined.

**New Brunswick, Restigouche Co.**, 8.5 km S of Saint Arthur, 47.8196°N, 66.7596°W, 14.VI.2006, R. P. Webster // Mixed forest, gravel bottomed pool near roadside (4, RWC). **Sunbury Co.**, Sheffield, Portobello Creek N.W.A. [National Wildlife Area], 45.8952°N, 66.2728°W, 18.VI.2004, R.P. Webster // Silver maple forest, u.v. light trap near marsh (1, RWC). **York Co.**, 45.8428°N, 66.7279°W, 2.VI.2003, R.P. Webster // Mixed forest, small pool on forest trail (1, RWC).

####### Distribution in Canada and Alaska.

BC, AB, SK, MB, ON, QC, **NB** (Bousquet et al. 2013).

###### Berosus peregrinus

Taxon classificationAnimaliaColeopteraHydrophilidae

(Herbst, 1797)

####### Material examined.

**New Brunswick, Carleton Co.**, Bell Forest, 46.2200°N, 67.7231°W, 13.VII.2004, K. Bredin, J. Edsall, & R. Webster // Mature hardwood forest, u.v. light trap (1, NBM); Meduxnekeag Valley Nature Preserve, 46.1888°N, 67.6762°W, 4. VII.2005, R. P. Webster // River margin, in flood debris (1, RWC); Meduxnekeag Valley Nature Preserve, 46.1941°N, 67.6830°W, 30.VI.2014, R. P. Webster // River margin with cobblestones, collected at night using headlamp (1, NBM; 1, RWC). **Kings Co.**, Hampton, Hampton Marsh at the Hammond River, 45.4787°N, 65.9007°W, 13.VII.2005 // R. P. Webster, river margin with sand/clay bottom (1, NBM; 2, RWC). **Queens Co.**, Grand Lake near Scotchtown, 45.8762°N, 66.1816°W, 9.VII.2006, R. P. Webster // Oak and maple forest, m.v. light (1, RWC); Bayard at Nerepis River, 45.4426°N, 66.3280°W, 20.VI.2008, R.P. Webster (1, RWC) . **Sunbury Co.**, Blissville, S Branch Oromocto River, 45.5996°N, 66.5604°W, 5.VII.2006, R. P. Webster // Gravel bottomed river in trailing vegetation (2, RWC). **York Co.**, Douglas, Nashwaaksis River at Rt. 105, 45.9850°N, 66.6900°W, 26.VI.2005, R. P. Webster // River margin in embayment with sand/gravel bottom, sun-exposed (1, RWC); Charters Settlement, 45.8395°N, 66.7391°W, 23.VII.2007, R.P. Webster // Mixed forest, m.v. light (1, RWC).

####### Distribution in Canada and Alaska.

ON, QC, **NB**, NS (Bousquet et al. 2013).

###### Berosus sayi

Taxon classificationAnimaliaColeopteraHydrophilidae

Hansen, 1999

####### Material examined.

**New Brunswick, Albert Co.**, Shepody N.W.A., Germantown Section, 45.7060°N, 64.7640°W, 16.VI.2004, R. P. Webster // Cattail and sedge marsh in small pool (1, RWC). **Gloucester Co.**, Miscou Island, 47.9081°N, 64.5907°W, 31.VII.2005, R. P. Webster // Shallow (15 cm) gravel pit pond with scattered grasses and sedges and gravel bottom (1, RWC). **Queens Co.**, Grand Lake near Scotchtown, 45.8762°N, 66.1816°W, 19.V.2003, V. Webster, M.-A. Giguère & R. Webster // Shallow lake margin among grasses (1, RWC); Grand Lake, Scotchtown, 45.8763°N, 66.1822°W, 16.VI.2013, R.P. Webster // Lake margin in shallow water (1, NBM); near Jemseg, 45.8237°N, 66.1393°W, 7.VI.2003 // Vernal pond in meadow, grass and sedge bottom (1, RWC); Rt. 105, N of Coytown, 45.8237°N, 66.1393°W, 17.VI.2013, R.P. Webster // Flooded meadow near seasonally flooded forest/marsh (1, NBM). **Sunbury Co.**, Maugerville, Portobello Creek N.W.A., 45.8992°N, 66.4248°W, 18.VI.2004, R. P. Webster // Silver maple forest near slow flowing river, black light trap (1, RWC). **York Co.**, Charters Settlement, 45.8352°N, 66.7330°W, 24.V.2003, R. P. Webster // mixed forest, margin of beaver pond among sedges (1, RWC); Charters Settlement, 45.8395°N, 66.7391°W, 24.V.2003, 7.IX.2007, 3.IX.2010, R. P. Webster // Mixed forest, u.v. light (2, NBM; 2, RWC); near Thomaston Corner, 45.637°N, 67.113°W, 13.IX.2003, V. Webster & R. Webster // Gravel pit pond (1, RWC).

####### Distribution in Canada and Alaska.

NT, BC, AB, SK, MB, ON, QC, **NB**, PE (Bousquet et al. 2013).

##### Tribe Laccobiini Houlbert, 1922

###### Paracymus despectus

Taxon classificationAnimaliaColeopteraHydrophilidae

(LeConte, 1863)

####### Material examined.

**New Brunswick, Queens Co.**, C.F.B. Gagetown, 45.7516°N, 66.1866°W, 3-15.VII.2013, C. Alderson & V. Webster // Old mixed forest with *Quercus rubra*, Lindgren funnel trap in canopy of *Quercus rubra* (1, AFC). **York Co.**, Canterbury, Browns Mtn. Fen [Eel River P.N.A.], 45.8967°N, 67.6343°W, 23.VI.2005, J. Edsall & R. Webster // Cedar swamp, in shaded moss-covered pools (4, RWC); same locality but 23.VI.2014, R.P. Webster // Calcareous cedar fen with shrubby cinquefoil, treading saturated sphagnum along moose trail (1, NBM; 1, RWC).

####### Distribution in Canada and Alaska.

AB, SK, MB, ON, QC, **NB** (Bousquet et al. 2013).

####### Comments.

Most specimens from New Brunswick were collected from saturated sphagnum moss in an open calcareous cedar fen. Smetana (1988) mentions that *Paracymus despectus* (LeConte) frequents shallow water with abundant organic debris but noted that little else was known about its habitat preferences.

#### Subfamily Chaetarthriinae Bedel, 1881

##### Tribe Chaetarthriini Bedel, 1881

###### Chaetarthria atra

Taxon classificationAnimaliaColeopteraHydrophilidae

(LeConte, 1863)

####### Material examined.

**New Brunswick, Carleton Co.**, Belleville, Meduxnekeag Valley Nature Preserve, 46.1942°N, 67.6832°W, 9.VI.2008, R.P. Webster // River margin, among cobblestones set in sand and fine gravel near water’s edge (2, RWC). **Queens Co.**, Bayard at Nerepis River, 45.4426°N, 66.3280°W, 30.V.2008, 25.VI.2010, R.P. Webster // River margin, under small rocks in gravel and in moist gravel (5, RWC)

####### Distribution in Canada and Alaska.

QC, **NB**, NS (Bousquet et al. 2013).

####### Comments.

In New Brunswick, adults of *Chaetarthria atra* (LeConte) were found on river margins among cobblestones set in fine sand and gravel, under small rocks, and in moist gravel at water’s edge. Little was previously known about its habitat preferences (Smetana 1988).

### Subfamily Enochrinae Short & Fikáček (2013)

#### Cymbiodyta acuminata

Taxon classificationAnimaliaColeopteraHydrophilidae

Fall, 1924

##### Material examined.

**New Brunswick, Albert Co.**, Shepody N.W.A., Germantown Section, 45.7056°N, 64.7642°W, 17.V.2004, R. P. Webster // Cattail and sedge marsh in marsh litter (1, RWC). **York Co.**, Charters Settlement, 45.8263°N, 66.7350°W, 5.V.2003, R. P. Webster // Sedge marsh, in pools among sedges and sphagnum moss (1, RWC).

##### Distribution in Canada and Alaska.

AK, YK, NT, BC, AB, SK, MB, ON, QC, **NB**, NS (Bousquet et al. 2013).

#### Cymbiodyta blanchardi

Taxon classificationAnimaliaColeopteraHydrophilidae

Horn, 1890

##### Material examined.

**New Brunswick, Albert Co.**, Caledonia Gorge P.N.A., 45.8380°N, 64.8484°W, 3.VII.2011, R.P. Webster // Near Turtle Creek, old-growth hardwood forest, mossy [spring-fed] seepage with some *Carex*, sifting saturated moss (1, RWC). **Carleton Co.**, Meduxnekeag Valley Nature Preserve, 46.1890°N, 67.6706°W, 8.VI.2005, R. P. Webster // Old-growth cedar stand, in saturated moss and debris in spring-fed seepage (7, RWC); [Jackson Falls] Bell Forest, 46.2210°N, 67.7210°W, 2.VI.2005, R. P. Webster // Mature hardwood forest, among small stones in spring-fed brook (2, RWC). **York Co.**, Charters Settlement, 45.8380°N, 66.7309°W, 14.V.2004, R. P. Webster // Mixed forest, small clear stream among gravel and stones (1, RWC).

##### Distribution in Canada and Alaska.

ON, QC, **NB** (Bousquet et al. 2013).

##### Comments.

Most specimens of *Cymbiodyta blanchardi* Horn from New Brunswick were found in saturated moss and debris in spring-fed seepages. Others were found among small stones in a spring-fed brook and among gravel and stones in a small clear stream. Smetana (1988) reported the species from similar habitats, including moss and algae on dripping cliffs. Little else is known about the biology of this species.

#### Cymbiodyta minima

Taxon classificationAnimaliaColeopteraHydrophilidae

Notman, 1919

##### Material examined.

**New Brunswick, Albert Co.**, Shepody N.W.A., Germantown Section, 45.7101°N, 64.7542°W, 17.V.2004, R. P. Webster // Spruce, fir, birch forest near large marsh, in leaf litter (1, RWC). **Sunbury Co.**, Sheffield, Portobello Creek N.W.A., 45.8965°N, 66.2725°W, 1.VIII.2004, R. P. Webster // Silver maple swamp near seasonally flooded marsh, u.v. light trap (1, RWC).

##### Distribution in Canada and Alaska.

BC, MB, ON, QC, **NB** (Bousquet et al. 2013).

#### Enochrus (Lumetus) hamiltoni

Taxon classificationAnimaliaColeopteraHydrophilidae

Horn, 1890

##### Material examined.

**New Brunswick, Gloucester Co.**, Caraquet near Rivière du Nord, 47.7949°N, 65.0903°W, 15.VIII.2003, R. P. Webster // Salt marsh, in small salt pond (2, NBM, 1, RWC); Maisonette Marsh, 47.8150°N, 65.0146°W, 30.VII.2005, R. P. Webster // Salt marsh, under litter on margin of salt pond (9, NBM; 2, RWC). **Kings Co.**, Plumweseep Salt Spring, 45.74326°N, 65.43819°W, 19.VI.2012, R.P. Webster & D. Sabine // Inland salt spring with clay margin with sparse vegetation (salt grass), splashing (1, RWC). **Madawaska Co.**, Loon Lake, 236 m elev., 47.7839°N, 68.3943°W, 21.VII.2010, R.P. Webster // Boreal forest, small lake surrounded by sedges, treading sedges near *Myrica gale* bushes (1, RWC). **Queens Co.**, near Jemseg [Grand Lake Meadows P.N.A.], 45.8237°N, 66.1393°W, 7.VI.2003, R.P. Webster // Vernal pond in meadow, grass and sedge bottom (4, RWC). **Restigouche Co.**, near Morin Bog, N of Kedgwick, 47.6828°N, 67.3148°W, 6.VI.2003, R. P. Webster // Black spruce forest, sedge marsh in large semi permanent flooded pool (1, RWC). **Sunbury Co.**, Maugerville, Portobello Creek N.W.A., 45.8992°N, 66.4248°W, 18.VI.2004, R. P. Webster // Silver maple forest near slow flowing river, black light trap (1, RWC). **York Co.**, Fredericton, 4.VI.1930, R. P. Gorham (1, AFC); Charters Settlement, 45.8248°N, 66.7220°W, 11.V.2003, 18.V.2003, R. P. Webster // Mixed forest, small eutrophic pond in dense vegetation (3, RWC).

##### Distribution in Canada and Alaska.

NT, BC, AB, SK, MB, ON, QC, **NB**, NS, PE, NF (Bousquet et al. 2013).

#### Enochrus (Methydrus) consors

Taxon classificationAnimaliaColeopteraHydrophilidae

(LeConte, 1863)

##### Material examined.

**New Brunswick, Queens Co.**, Grand Lake Meadows P.N.A., 45.8227°N, 66.1209°W, 24.VIII-3.IX.2010, R.P. Webster // Old silver maple forest with green ash and seasonally flooded marsh, Lindgren funnel trap (1, RWC); same locality data, forest type and collection method but 5-17.VIII.2011, M. Roy & V. Webster (1, RWC). **Restigouche Co.**, Otter Brook Fen, 47.9337°N, 68.0532°W, 30.VII.2012, R. Webster & M. Turgeon // *Carex* marsh on lake margin, treading vegetation (1, RWC).

##### Distribution in Canada and Alaska.

ON, QC, **NB** (Bousquet et al. 2013).

#### Enochrus (Methydrus) consortus

Taxon classificationAnimaliaColeopteraHydrophilidae

Green, 1946

##### Material examined.

**New Brunswick, Queens Co.**, Grand Lake near Scotchtown, 45.8762°N, 66.1816°W, 9.VII.2006 // R. P. Webster, oak and maple forest, m.v. light (2, RWC). **Sunbury Co.**, Maugerville, Portobello Creek N.W.A., 45.8992°N, 66.4248°W, 18.VI.2004, R. P. Webster // Silver maple forest near slow flowing river, black light trap (1 [male dissected], RWC). **York Co.**, Charters Settlement, 45.8395°N, 66.7391°W, 17.VI.2004, R.P. Webster // Mixed forest, m.v. light (1, RWC)

##### Distribution in Canada and Alaska.

MB, ON, **NB** (Bousquet et al. 2013).

#### Enochrus (Methydrus) pygmaeusnebulosus

Taxon classificationAnimaliaColeopteraHydrophilidae

(Say, 1824)

##### Material examined.

**New Brunswick, York Co.**, Charters Settlement, 45.8395°N, 66.7391°W, 23.VII.2007, R. P. Webster // Mixed forest, u.v. light (1, RWC).

##### Distribution in Canada and Alaska.

MB, ON, QC, **NB** (Bousquet et al. 2013).

### Subfamily Sphaeridiinae Latreille, 1802

#### Tribe Megasternini Mulsant, 1844

##### Cercyon (Cercyon) cinctus

Taxon classificationAnimaliaColeopteraHydrophilidae

Smetana, 1978

###### Material examined.

**New Brunswick, York Co.**, Charters Settlement, 45.8395°N, 66.7391°W, 23.VII.2007, 20.VIII.2011, R. P. Webster // Mixed forest, m.v. light (2, RWC); Douglas, Currie Mountain, 45.9832°N, 66.7564°W, 24.VI–9.VII.2013, C. Alderson & V. Webster // Old *Pinus strobus* stand, Lindgren funnel trap in canopy of *Pinus strobus* (1, RWC).

###### Distribution in Canada and Alaska.

NT, BC, AB, SK, MB, ON, QC, **NB** (Bousquet et al. 2013).

##### Cercyon (Cercyon) herceusfrigidus

Taxon classificationAnimaliaColeopteraHydrophilidae

Smetana, 1978

###### Material examined.

**New Brunswick, Albert Co.**, Shepody N.W.A., Germantown Section, 45.7056°N, 64.7642°W, 17.V.2004, R. P. Webster // Cattail and sedge marsh, in marsh litter (1, RWC); Caledonia Gorge P.N.A., 45.7930°N, 64.7764°W, 1.VII.2011, R.P. Webster // Small rocky clear-cold river (Caledonia Creek), sifting drift material (tree bud material) near eddy area (1, RWC). **Carleton Co.**, Two Mile Brook Fen, 46.3619°N, 67.6733°W, 6.V.2005, R.P. Webster // Cedar forest/swamp, in moist sphagnum (1, RWC). **Queens Co.**, W of Jemseg near “Trout Creek” [Grand Lake Meadows P.N.A.], 45.8227°N, 66.1240°W, 9.V.2004, R.P. Webster // Silver maple swamp, sifting litter at base of large tree (1, RWC). **Sunbury Co.**, Maugerville, Portobello Creek N.W.A., 45.8992°N, 66.4248°W, 5.VI.2004, R. P. Webster // Silver maple forest, margin of small pond in leaf litter (1, RWC); Gilbert Island, 45.8770°N, 66.2954°W, 12-29.VI.2012, C. Alderson, C. Hughes & V. Webster // Hardwood forest, Lindgren funnel trap 1 m high under *Juglans cinerea* (1, RWC). **York Co.**, Fredericton, at Saint John River, 45.9588°N, 66.6254°W, 4.VII.2004, R.P. Webster // River margin, in drift material (mostly maple seeds) (1, RWC); Charters Settlement, 45.8395°N, 66.7391°W, 15.IV.2004, R. P. Webster // Mixed forest, in leaf litter near small stream (2, RWC); same locality data and collector but 9.IV.2005 // Residential lawn among lawn grass adjacent to garden (1, RWC).

###### Distribution in Canada and Alaska.

AK, YT, NT, BC, AB, SK, MB, ON, QC, **NB** (Bousquet et al. 2013).

##### Cercyon (Dicyrtocercyon) ustulatus

Taxon classificationAnimaliaColeopteraHydrophilidae

(Preyssler, 1790)†

###### Material examined.

**New Brunswick, Sunbury Co.**, Gilbert Island, 45.8770°N, 66.2954°W, 18-28.V.2012, C. Alderson, C. Hughes & V. Webster // Hardwood forest, Lindgren funnel trap 1 m high under *Tilia americana* (1, RWC).

###### Distribution in Canada and Alaska.

QC, **NB** (Bousquet et al. 2013).

## Supplementary Material

XML Treatment for Helophorus (Kyphohelophorus) tuberculatus

XML Treatment for Helophorus (Rhopalelophorus) marginicollis

XML Treatment for Helophorus (Rhopalelophorus) oblongus

XML Treatment for Hydrochus subcupreus

XML Treatment for Berosus fraternus

XML Treatment for Berosus peregrinus

XML Treatment for Berosus sayi

XML Treatment for Paracymus despectus

XML Treatment for Chaetarthria atra

XML Treatment for Cymbiodyta acuminata

XML Treatment for Cymbiodyta blanchardi

XML Treatment for Cymbiodyta minima

XML Treatment for Enochrus (Lumetus) hamiltoni

XML Treatment for Enochrus (Methydrus) consors

XML Treatment for Enochrus (Methydrus) consortus

XML Treatment for Enochrus (Methydrus) pygmaeusnebulosus

XML Treatment for Cercyon (Cercyon) cinctus

XML Treatment for Cercyon (Cercyon) herceusfrigidus

XML Treatment for Cercyon (Dicyrtocercyon) ustulatus
